# Twelve-Day Reinforcement-Based Memory Retention in African Cichlids (*Labidochromis caeruleus*)

**DOI:** 10.3389/fnbeh.2016.00157

**Published:** 2016-08-17

**Authors:** Erica Ingraham, Nicole D. Anderson, Peter L. Hurd, Trevor J. Hamilton

**Affiliations:** ^1^Department of Psychology, MacEwan UniversityEdmonton, AB, Canada; ^2^Neuroscience and Mental Health Institute, University of AlbertaEdmonton, AB, Canada

**Keywords:** memory, long-term, operant conditioning, learning and memory, cichlid fish, food reinforcement

## Abstract

The formation of long-term memories for food sources is essential for the survival of most animals. Long-term memory formation in mammalian species has been demonstrated through a variety of conditioning tasks, however, the nature of long-term memory in fish is less known. In the current study, we explored whether African cichlids (*Labidochromis caeruleus*) could form memories for food-reinforced stimuli that last for 12 days. During the training sessions, fish were reinforced for approaching an upward drifting line grating. After a rest period of 12 days, fish demonstrated a significant preference for the upward drifting grating. To determine whether this preference could also be reversed, fish were then reinforced for approaching a downward drifting line grating after a 20-day rest period. When tested 12 days later, there were no significant differences in preference for either stimulus; however, following a second training period for the downward stimulus, there was a significant preference for the downward drifting grating. This suggests that cichlids are able to form reversible discrimination-based memories for food-reinforced stimuli that remain consolidated for at least 12 days.

## Introduction

Long-term memories for biologically relevant information are an essential mechanism for survival in a wide variety of animals. Memories that span on the order of years have been demonstrated in young monkeys (Murai et al., 2011) and on the order of decades in bottle-nosed dolphins (Bruck, 2013) and elephants (McComb et al., 2000). Although the advantages of a reliable long-term memory system are advantageous there are also costs that arise with the possession of long-term memory stores. One cost, for example, is the ability to retain a faithful representation of complex information for a prolonged period of time, as this will require additional neural mechanisms (Dukas, 1999). This is demonstrated in food-storing birds, for example, who possess larger hippocampi relative to those in non-storing birds (e.g., Krebs et al., 1989; Sherry et al., 1989; Krebs, 1990). Moreover, previously formed memories can interfere with the learning of new information, requiring mechanisms to extinguish memory traces that are no longer necessary (Dukas, 1999). Given these costs of long-term memory storage, one might expect a reduction in long-term memory abilities in animals with limited neural resources.

On average, the fish brain is approximately 1/15 of the relative size of analogous brains in birds or mammals (Helfman et al., 2009: p. 54). Because of the relative size of the fish central nervous system there have been assumptions that fish have minimal cognitive skills, which in turn has led to less research on cognition in fish relative to rodents. However, even given their limited neural resources, new evidence is arising that suggests that a multitude of fish species are surprisingly intelligent as assessed by standard measures and can perform surprisingly complex tasks (for reviews, see Braitewaite, 2005; Brown, 2015). For example, social learning was demonstrated in salmon (Brown and Laland, 2002) and guppies (Laland and Williams, 1997). The ability to judge numerosity has been observed in mosquitofish (Agrillo et al., 2009) and guppies (Bisazza et al., 2014). Tool use behaviors were even observed in wrasse (Jones et al., 2011), catfish (Armbrust, 1958) and cichlids (Timms and Keenleyside, 1975; Keenleyside and Prince, 1976). In studies that focus on learning abilities in fish, goldfish (*Carassius auratus*) are able to discriminate between different food-reinforced stimuli and have been successfully trained in spatial learning paradigms (Rodriguez et al., 1994; Arthur and Levin, 2001; Frech et al., 2012). Wild-caught damselfish (*Pomacentrus amboinensis*) also have associative learning capabilities (Siebeck et al., 2009). Given the observation of these complex behaviors in fish, one would suspect that other cognitive skills, such as memory, might be better than originally thought.

Evidence for long-term memory in fish can be demonstrated through a variety of conditioning tasks. Zebrafish (*Danio rerio*), for example, can remember the correct location of food reinforcement in a T-maze with colored arms (Colwill et al., 2005). This was demonstrated using both extinction and reversal trials which suggest that fish were retaining a memory for the original color-food association for some trials following the initial acquisition (Colwill et al., 2005). There is also evidence for one-trial object recognition (novel object recognition) learning in zebrafish lasting from 5 min (May et al., 2015) to 24 h (Braida et al., 2014; Lucon-Xiccato and Dadda, 2014), and least 24 h with a shock-based task (Blank et al., 2009). Zebrafish also have the capacity for episodic-like memory (Hamilton et al., 2016). Fewer studies, however, explore the duration of long-term memories in fish. Memory span was previously demonstrated in zebrafish using a spatial alteration task and shown to last at least 10 days (Williams et al., 2002). Rainbow fish (*Melanotaenia duboulayi*) were shown to have a reduced latency to escape an aversive stimulus almost 1 year after initial exposure to the experimental set up when compared to fish who had never been placed in the testing arena (Brown, 2001).

We evaluated whether “electric yellow” cichlid fish (*Labidochromis caeruleus*) would create and maintain a reinforcement-based memory over a period of 12 days. Cichlids were reinforced to respond to a drifting line grating pattern that was presented at one end of the training arena, and memory for the pattern was assessed 12 days after training to establish whether a memory trace for the trained pattern existed. We also conducted reversal training, where fish were trained to respond to the opposite drifting pattern in order to establish whether any observed effects were due to the formation of a memory trace and not to an inherent sensory bias. Our results suggest that cichlids are capable of forming reinforcement-based memories for at least a period of 12 days.

## Materials and Methods

### Animals and Housing

Electric yellow cichlids (*Labidochromis caeruleus*; *n* = 7) ranging in age from 0.5 to 1.5 years were housed together in a 60 L tank (30 × 60 × 40 cm) with transparent walls. This community housing tank contained a submersible heater, sand substrate, eight rock hiding spots, and two air-stones that constantly created bubbles in the water. Water (10%) was changed once a day using dechlorinated tap water. Fish were housed with a 12 h light/dark cycle, with lights switched on at 8 AM and off at 8 PM. Fish were normally fed with pelleted food (New Life Spectrum 3 mm Sinking Pellets, New Life International Inc., FL, USA) once a day. Water quality measures daily included temperature, pH and dissolved oxygen. Weekly water quality measures included that of ammonia, nitrates, nitrites, alkalinity, hardness and conductivity. This research was approved by the MacEwan University Animal Research Ethics Board, protocol number 05-12-13, under Canadian Council for Animal Care (CCAC) guidelines.

### Stimuli and Apparatus

**Figure 1:**
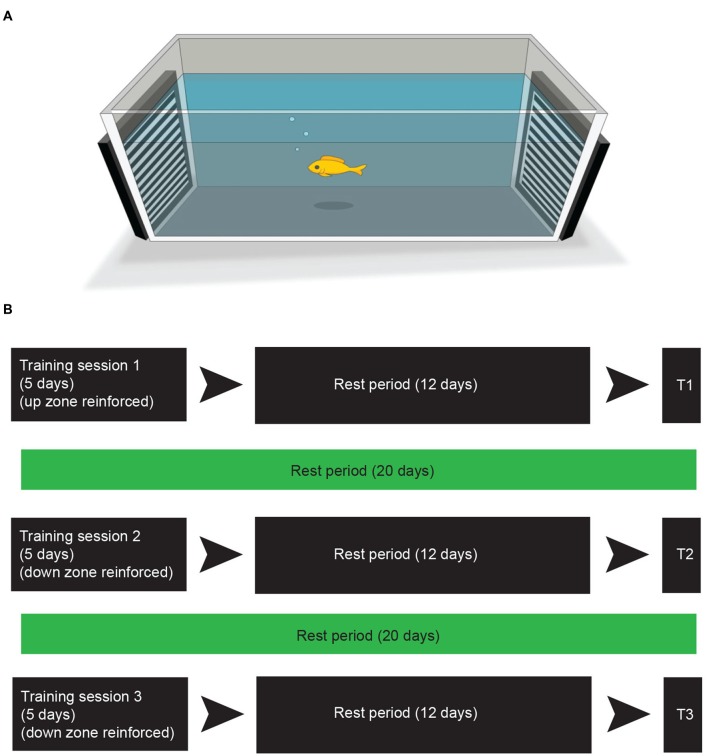
**Training leads to preference for reinforcement zone. (A)** The apparatus used for training and testing. Computer screens on either end of the tank display drifting line gratings. See “Materials and Methods” Section for training and testing procedure. **(B)** The experimental training and testing schedule. In the first round of training fish were individually placed into the arena **(A)** and reinforced for moving into the “up zone” (within 25 cm of the moving stimulus) for 20 min per fish. This training took place 3 times in a 5-day period. The fish were then placed back into their community housing tank for 12 days (rest period). Test period 1 (T1) took place the day after the rest period. Following T1 the fish remained in their community housing tank for 20 days. The subsequent day training session 2 began and was identical to training session 1 with the exception of the reinforcement, which was switched to the “down zone”. Test period 2 (T2) was performed after a 12 day rest period. The third round of training and testing (T3) was identical to the second round.

The training and testing sessions were conducted in a rectangular arena (91 × 46 × 31.5 cm) that was filled with 8.5 cm of water (26–29°C). Water was aerated with oxygen between training sessions. The stimuli used for reinforcement were two separate drifting sinusoidal gratings that were presented full-screen on two Dell laptop screens (1600 × 900 resolution) placed at each end of the arena (see Figure 1A). The gratings were horizontally oriented, where one grating drifted in an upward direction and the other drifted in a downward direction. The placement of the drifting gratings on each end of the arena was randomized for every fish prior to the beginning of the training. Spatial frequency and drift velocity of the gratings were held constant at 1.12 cycles/cm and 2.67 cm/s respectively. Michelson contrast was held constant at 0.9895. Gratings were generated using GNU Octave and presented using the Psychophysics toolbox (Brainard, 1997; Pelli, 1997) in the Linux Ubuntu 12.04 (64 bit) environment.

### Training Procedure

Fish were not fed the day prior to training to increase salience of the food-reinforcer. On the training day, each fish was netted and directly placed into the center of the experimental apparatus facing the long axis. Fish were trained individually. Thirty seconds after being placed into the arena, fish received a single food pellet each time they moved within 25 cm of the target stimulus (i.e., the training zone), which was marked on the outside of the tank and was not visible to the fish. The experimenter administered reinforcers by dropping a food pellet (New Life Spectrum 3 mm Sinking Pellets, New Life International Inc., FL, USA) into the training zone. Fish were given one food pellet for each training zone entry in which they remained for 5 s. They were given an additional food pellet if they remained in the training zone for a span of 1 min. For each fish the training session lasted for 20 min. Both drifting gratings were presented for the entire session. Training took place once per day between 10 AM and 3 PM and occurred three times during a 5-day period. All training sessions took place before daily feeding.

For the first training period, the training zone was assigned to be the upward drifting grating (i.e., the upward training zone). After a 12-day rest period in the community housing tank, fish were tested following the procedure described below (test period 1; see Figure 1B for an experimental time schedule). Reversal training took place 20 days later. For reversal training, the training procedure was repeated but the training zone was assigned to be the downward drifting grating (i.e., the downward training zone). Fish were again tested 12 days later (test period 2). An additional downward training period took place 20 days later followed by a third round of testing 12 days later (testing period 3).

### Testing Procedure

Tests were conducted using the same arena and stimuli described above, but did not involve the food reinforcement. Testing trials were conducted in a different room to eliminate any potential cues that may have led to a spatial bias. Fish were deprived of food for 1 day prior to testing. Each fish was individually placed in the center of the arena and released facing the short axis (i.e., not facing either moving line grating). During testing, movement was recorded using the differencing method in Ethovision XT motion tracking software for 5 min (version 7.0, Noldus, VA, USA) with the camera mounted 1 m directly above the test apparatus. Trials began immediately after fish were placed in the testing arena. Dependent variables included time spent in training zones (upward or downward), number of zone transitions, and average velocity.

### Statistical Analysis

Data were analyzed for the full 5 min trial. All data were assessed for normality using the Shapiro-Wilk normality test. Time spent in reinforced zones (referred to as “training zones” above) was analyzed using an unpaired *t*-test for normally distributed data or using Mann-Whitney U for nonparametric data. Velocity and zone transitions (number of times a fish crossed the mid-line of the short axis of the arena) were analyzed with a one-way analyses of variance (ANOVA) or Kruskal-Wallis test for non-parametric data (fish were not individually tagged so repeated measures ANOVA was not performed). Data were analyzed using GraphPad Prism 6 (GraphPad Software Inc., La Jolla, CA, USA) and were presented as mean values ± SEM.

## Results

**Figure 2:**
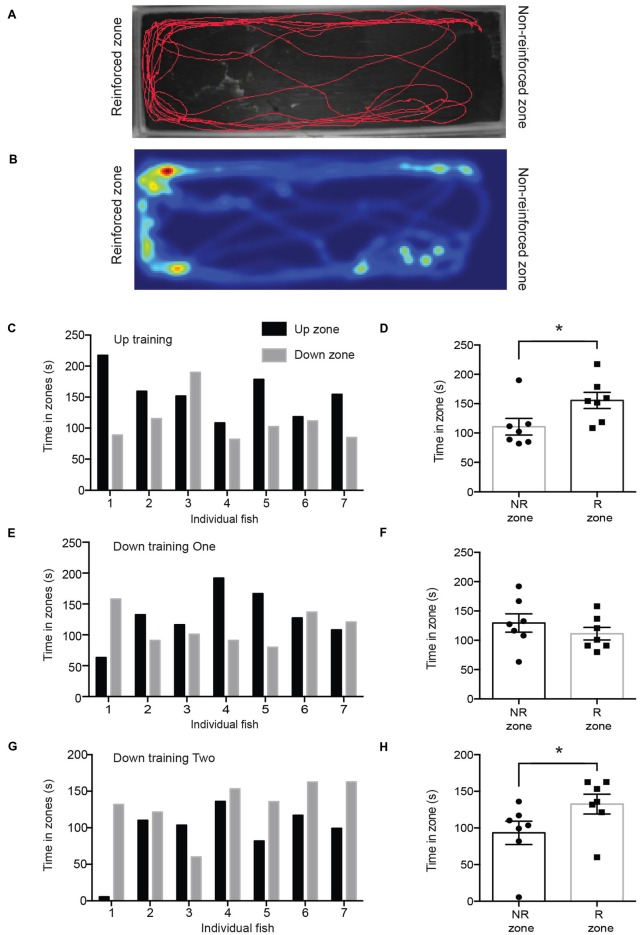
**(A)** Trackplot of representative fish during one trial of testing period 1. **(B)** The heatmap is a colored representation of the same fish and is proportional to the time spent in each pixel. **(C)** During training session 1, the up zone was reinforced and in test period 1 all fish except for one spent more time in the up zone. **(D)** Fish spent significantly more time in the reinforced zone (R zone) than in the non-reinforced zone (NR zone; reinforced zone; 155.5 ± 13.8 s; non-reinforced zone; 110.7 ± 14.1 s; *p = 0.0423). **(E)** During training session 2 the down zone was reinforced. This graph shows the time spent in the up or down zone during test period 2. **(F)** Fish did not spend significantly more time in the reinforced zone (R zone) compared to that in the non-reinforced zone (NR zone; reinforced zone; 129.5 ± 15.7 s; non-reinforced zone; 111.2 ± 10.7 s; *p* = 0.1774) in test period 2. **(G)** During test period 3, after the down zone had been reinforced a second time, all fish except for one spent more time in the down zone. Note that the order of testing on each day was random and the individual fish number does not correspond to Panel **(C)** or **(E)**. **(H)** Fish spent significantly more time in the reinforced zone than in the non-reinforced zone (reinforced zone; 132.6 ± 13.45 s; non-reinforced zone; 93.4 ± 16.0 s; *p = 0.0265) in test period 3. Values are mean ± SEM. *p < 0.05.

We first tested fish 12 days after they had been reinforced for entering the upward training zone (“up zone”; Figures 2A,B). Every fish except for one spent more time in the up zone where it had been reinforced (test period 1; Figure 2C). There was a significant difference in time spent in the reinforced zone compared to the non-reinforced zone (Figure 2D; reinforced zone; 155.5 ± 13.8 s; non-reinforced zone; 110.7 ± 14.1 s; *p* = 0.0379). After this experiment, the same fish were trained with the opposite stimulus (downward training zone) and tested 12 days later. Following this reversal training, fish did not show a significant preference for either zone (reinforced zone; 111.2 ± 10.7 s; non-reinforced zone; 129.5 ± 15.7 s; *p* = 0.1774; Figures 2E,F). We then performed a second round of training to the downward moving stimulus (this was the third training period) and retested their location preference. During test period 3, fish spent significantly more time in the downward training zone (“down zone”), which had been reinforced (Figures 2G,H; reinforced zone; 132.6 ± 13.5 s; non-reinforced zone; 93.4 ± 16.0 s; *p* = 0.0265). The number of zone transitions and velocity did not differ between any of the testing periods (Table 1).

**Table 1:** **Velocity and zone transitions during each testing period**.

	Test 1	Test 2	Test 3
Velocity (cm/s)	10.24 ± 1.61	8.37 ± 0.85	7.60 ± 0.65
Zone transitions	11.29 ± 3.12	9.00 ± 1.20	10.14 ± 0.73

*There were no significant differences in velocity during any of the testing trials [H_(N = 21)_ = 3.391, P = 0.1835] nor were there any differences in zone transitions [H_(N = 21)_ = 0.5344, P = 0.7655]*.

## Discussion

Our results indicate that electric yellow (*Labidochromis caeruleus*) cichlids are able to develop reinforcement-based memories for different moving visual patterns and maintain those memories over a period of at least 12 days. In order to develop these memories, fish needed to develop an association between the stimuli and the food rewards, and also discriminate between the upward and downward drifting gratings. Fish were able to learn to associate the upward moving stimulus with food after only three short training sessions (per fish) and were able to create a lasting memory that was apparent when they were tested 12 days later (Figures 2C,D). Interestingly, following the first round of down zone training (reversal training), fish did not show a preference for the down zone; however, they also no longer exhibited a preference for the up zone. Following an additional training period for the down zone, fish fully reversed their preference for the appropriate reinforced zone (Figures 2G,H). This suggests that the memory trace for the initial training session interfered with the development of a new association in the first reversal test session, even though there was a 33-day delay between the last upward training session and the first reversal test session. Following the second round of training on the downward moving stimulus, fish exhibited a preference for the down zone. These results demonstrate that fish are able to form and maintain reinforcement-based memories, and also create new memories for previously non-reinforced stimuli.

Previous research also demonstrated that cichlids can form reinforcement-based memories (Mark and Maxwell, 1969; Schluessel et al., 2012; Gierzewski et al., 2013). However, these studies did not probe the time-span of the memory trace that was consolidated in the fish. Moreover, the nature of the stimuli that we used was critically different from the stimuli used in these studies. The stimuli that were used in previous research consisted of stationary shapes and patterns. Our stimuli were purely defined through motion. Across the animal kingdom, sensitivity to features such as orientation, color, and form can vary widely from species to species. All visual organisms, on the other hand, are sensitive to motion. Given the biological relevance of motion as a visual cue, we reasoned that motion would be a salient stimulus that fish could develop strong memory traces for.

The full capacity of the cichlid memory span is not currently known, but a minimum of 12 days, as seen in our current study, is not unreasonable. The possibility that previous memory for the up-zone training in the fish interfered with reversal training also suggests that the memory span for the initial food location might be as long as 33 days. This time span is consistent with the memory time spans that have been determined in other fish species. Zebrafish (*Danio rerio*) have been shown to maintain memory over a period of 10 days when tested using a spatial alteration task (Williams et al., 2002). Similarly, Paradise fish (*Macropodus opercularis*) exhibit reduced exploratory behavior during the second encounter with a novel fish 3 months after the initial exposure, suggesting an implicit memory span of at least 3 months (Csányi et al., 1989). Nilsson et al. (2007) demonstrated that Atlantic cod (*Gadus morhua*) were able to maintain memory for feeding locations 3 months after training. Rainbow fish exhibit faster escape times from an aversive stimulus 11 months after initial exposure and training to the stimulus (Brown, 2001). A critical difference between the studies by both Brown (2001) and Nilsson et al. (2007), and our study is that the former studies used paradigms that trained and tested groups of fish at a time. Under these circumstances, social learning may have enhanced the learning capabilities of the fish subjects. Fish were tested individually in our study, thereby allowing us to investigate memory abilities without potentially confounding social cues.

Establishing memories of food locations is essential for survival. It is especially important for animals to be able to learn not just about new food locations but also about their relative profitability (Warburton, 2003). Optimal foraging requires that alternative food patches be evaluated with respect to their profitability; a patch should be abandoned when it is more profitable to move to another one. The length of time Bluegill sunfish stay on a foraging patch depends upon what they have learned about the profitability of other patches (Wildhaber et al., 1994). It is inefficient for an animal to maintain memory for a food source once it is no longer available, or offers measly rewards. An individual who finds enough food to stay alive will nonetheless be far less fit than a competitor individual who consistently feeds more efficiently; that competitor will have more time to watch for predators, find a better mate, and defend their offspring and territory (Hamilton, 2010). Plasticity in memory is the key to this efficiency and can be demonstrated using serial reversal learning paradigms. These paradigms will help us to understand whether or not an animal is easily able to switch its preference to a new food location after the initial location no longer provides food. Warburton (1990) discovered that goldfish initially alternated between spatial locations when the food source was no longer in a known position but following this reversal period, fish showed a decreased latency to find the food source in the new location. Mattioli et al. (1997) also used a reversal period and found that goldfish were quickly able to learn about a new food location. Thus, these studies, as well as ours, demonstrate that fish are capable of learning to recall stimuli associated with the presence of food as well as learn a new location that now contains a more profitable source of food.

## Author Contributions

EI, NDA, and TJH conceived and designed the study; EI performed the study; EI and TJH analyzed the data; all authors wrote the article.

## Conflict of Interest Statement

The authors declare that the research was conducted in the absence of any commercial or financial relationships that could be construed as a potential conflict of interest.
